# Preclinical pharmacology and toxicology evaluation of an anti-CD52 monoclonal antibody produced by perfusion fermentation process

**DOI:** 10.1093/jimb/kuab078

**Published:** 2021-10-20

**Authors:** Yanchao Wang, Chen Zheng, Chao Zhuang, Qiang Fu, Jinyan Qin, Baohong Zhang, Yanling Bian, Nianmin Qi, Jianwei Zhu

**Affiliations:** Engineering Research Center of Cell and Therapeutic Antibody, MOE, School of Pharmacy, Shanghai Jiao Tong University, 800 Dongchuan Road, Shanghai 200240, China; Shanghai Taiyin Biotechnology Co., Ltd., 781 Cailun Road, Zhangjiang Hi-tech Park, Shanghai 201203, China; Shanghai Taiyin Biotechnology Co., Ltd., 781 Cailun Road, Zhangjiang Hi-tech Park, Shanghai 201203, China; Shanghai Taiyin Biotechnology Co., Ltd., 781 Cailun Road, Zhangjiang Hi-tech Park, Shanghai 201203, China; Shanghai Taiyin Biotechnology Co., Ltd., 781 Cailun Road, Zhangjiang Hi-tech Park, Shanghai 201203, China; Engineering Research Center of Cell and Therapeutic Antibody, MOE, School of Pharmacy, Shanghai Jiao Tong University, 800 Dongchuan Road, Shanghai 200240, China; Engineering Research Center of Cell and Therapeutic Antibody, MOE, School of Pharmacy, Shanghai Jiao Tong University, 800 Dongchuan Road, Shanghai 200240, China; Shanghai Taiyin Biotechnology Co., Ltd., 781 Cailun Road, Zhangjiang Hi-tech Park, Shanghai 201203, China; Engineering Research Center of Cell and Therapeutic Antibody, MOE, School of Pharmacy, Shanghai Jiao Tong University, 800 Dongchuan Road, Shanghai 200240, China

**Keywords:** Alemtuzumab, CD52, Biosimilar, Leukemia, Preclinical

## Abstract

Anti-cluster of differentiation 52 (CD52) monoclonal antibody (mAb) has been employed in the treatment of chronic lymphoblastic leukemia and multiple sclerosis. Previously we developed a perfusion process to produce the biosimilar mAb named “Mab-TH.” A series of quality assessments was conducted in the fields of structural identification, purity analysis, and activity measurement. After these quality researches, this report laid emphasis on preclinical pharmacology and toxicology evaluation. Mab-TH was characterized in biological, pharmacological, and toxicological properties in comparison with the original drug, alemtuzumab. Binding activity and immune-dependent toxicity as *in vitro* activity were evaluated. Severe immunodeficient mice transplanted with a human leukemia cell line were also used as an *in vivo* pharmacological model and a 4-week repeated dosing study in cynomolgus monkeys was conducted to evaluate the safety differences. Our results demonstrated that Mab-TH, the anti-CD52 antibody generated by a perfusion process, had high similarity in *in vitro* and *in vivo* activities compared with alemtuzumab in relevant preclinical models. The results supported it as a biosimilar candidate for clinical evaluation.

## Introduction

CD52 antigen is a lipid-anchored glycoprotein expressed on the surface of lymphocytes, monocytes, and macrophage (Hale, 2001). Alemtuzumab (under the trade name Campath), the humanized anti-CD52 monoclonal antibody (mAb), has already been approved for the treatment of resistant chronic lymphocytic leukemia (Hillmen et al., 2007; Keating et al., 2002). The antibody belongs to the Immunoglobulin G (IgG)) 1 kappa subgroup, with a molecule weight of 150 kDa. The whole antibody was composed of two light chains (214 amino acids) and two heavy chains (450 amino acids) with disulfide bond connection (Riechmann et al., 1988). Compared with traditional chemotherapy agents (alkylating agents and purine analogs), alemtuzumab could specifically recognize the CD52 antigen by its Fab region and induce lymphocyte lysis by complement-dependent cytotoxicity (CDC) (Zent et al., 2008) and antibody-dependent cytotoxicity (ADCC) (Golay et al., 2004), with high efficacy and low toxicity.

Fed-batch culture has been dominant in the large-scale industrial production of recombinant therapeutic proteins (Pollock et al., 2013; Siganporia et al., 2014; Zhu, 2012). This process is simple for operation, but a high density of cell degradation material present in the fermenter will cause problems such as aggregation and low productivity (Cromwell et al., 2006). In our previous work, we had created a perfusion process to produce anti-CD52 mAb (named “Mab-TH”). In the culture period of 35 days, medium with products was perfused through the bioreactor, while cells were retained or recycled back. This process offered consistent and steady culture conditions, allowed rapid removal of products, and gained a much higher productivity than the fed batch process. A series of quality researches has been conducted in the fields of peptide mapping, aggregation analysis, and CDC activity (Zhuang et al., 2017). The results showed that the final product of Mab-TH contained less impurity, higher effective configuration, and higher cytological activity compared with that from the fed-batch process (Zhuang et al., 2017).

Biological products with changes in the manufacturing process compared with the original products are defined as “biosimilar products” according to the regulations published by the Chinese National Medical Products Administration (NMPA). A biosimilar has to demonstrate a similar safety profile and demonstrate the same efficacy as the originator (Socinski et al., 2015). To ensure similarity of the structure-to-function relationship between Mab-TH and alemtuzumab, we analyzed their specific binding to CD52 and *in vitro* responses through CDC and ADCC. Severe combined immunodeficient (SCID) mice transplanted with a human leukemia cell line were also used as an *in vivo* pharmacological model. As CD52 is only expressed in old-world primates (Perry et al., 1992), only a part of cynomolgus monkeys were chosen for safety and toxicology research. Our results demonstrated that Mab-TH has highly comparable activities to alemtuzumab in relevant preclinical models, warranting a clinical evaluation of their biosimilarity.

## Materials and Methods

### Production of Mab-TH by Perfusion Process

The production of Mab-TH was described in our previous work (Zhuang et al., 2017), with some modification. The antibody was produced by a recombinant Chinese hamster ovary (CHO) cell line (TH6 cells) with Dynamics serum free medium (Thermo Fisher, USA). The culture process was performed in a 15 l bioreactor (Applikon, the Netherlands) at a final working volume of 12 l. An inoculum density of 0.5e6 to 1e6 cells/ml was employed and the dissolved oxygen concentration was at 40–60%. The pH of the fermentation broth was maintained at 6.9–7.1 by adding either CO_2_ or NaHCO_3_. The temperature was set at 37°C during the growth period and decreased to 35°C in the stationary period. A gravity settler was used for cell retention with a working volume of 12 l. The perfusion process was started on Day 5 and ended on Day 30.

Around 200 l cell culture supernatant was treated at one time by a three-step purification. First, it was inflown into a 1020 cm^2^ deep filter (the 3M corporation, USA), with the inlet pressure less than 0.15 Mpa. Then, the harvested liquid was concentrated by ultrafiltration with a 50 kDa membrane package (Sartorius, Germany) and passed through a 0.45 μM bag filter to obtain the clear concentrate (Sartorius, Germany). Finally, it was purified by protein-A affinity chromatography using an Mabselect SuRe column (GE Healthcare, USA).

### *In Vitro* Pharmacological Assessments

#### Binding assay

The binding assay was mainly based on the method reported by Peppy Rebello, with some modification (Rebello & Hale, 2002). MC/CAR cells (American Type Culture Collection [ATCC], USA) were chosen as the target cells with a high expression of CD52. The cells were harvested and washed by centrifugation three times and resuspended by a wash buffer (phosphate buffer [PBS] with 2% bovine serum albumin [BSA] and 1% sodium azide) at a cell concentration of 5e6 cells/ml. Ten microliters of Mab-TH antibody and alemtuzumab (Genzyme Corporation, USA) at concentrations 0.25–1000 μg/ml was mixed with 100 μl of cells. The mixture was incubated on ice for 1 hr and then washed three times by centrifugation at 1200 rpm and resuspension. Fluorescent reagent fluoresceine isothiocyanate (FITC)-labeled rabbit antihuman IgG (Sigma-Aldrich, USA) was then added and the mixture was incubated on ice for another hour. The cells were rewashed, resuspended by assay buffer (PBS with 2% BSA and 1% paraformaldehyde), and stored at 4°C until flow cytometry analysis.

The flow cytometry BD Accuri C6 (Becton Dickinson and Company, USA) was used to analyze the results. A region was set on the cytogram of forward versus sideways scatter to select the cells. A typical lymphocyte region was enclosed as the target gate in FACSan. Ten thousand events were collected for each sample. A histogram of the green fluorescence of the cells in this region was printed. The median fluorescence intensity (MFI) of targeted cells in the histogram was computed and the correspondence between the MFI and antibody concentration was used to calculate the EC_50_ value.

#### Complement-mediated lysis

Viability indicators such as propidium iodide and annexin V are often used in CDC assays (Ambrose et al., 2009; Zent et al., 2004), but their low sensitivity to reflect cell damage limits their application. Therefore, the ATP chemiluminescence reagent Promega G7571 (Promega, USA) was chosen as the indicator in our method. In 96-well plates, 20 000 cells were plated at a volume of 50 μl in the presence of 25 μl antibody at a concentration range from 0.25 to 1000 μg/ml. Twenty-five microliters of 25% human serum solution was added and the cells were incubated for 2 hr at 37°C. Finally, 100 μl G7571 reagent was added and the plates were read in a luminescence meter (Biotek, USA). EC_50_ was calculated from the correspondence between the luminescence value and the antibody concentration.

#### Antibody dependent cellular cytotoxicity

Antibody-dependent cellular cytotoxicity (ADCC) was characterized according to the lactate dehydrogenase (LDH) secreted in the lysis process. Twenty thousand target cells (MC/CAR) were cultivated with different concentrations of tested antibody. After overnight reaction, 20 000 effect cells natural killer (NK92 cells) (Chempartner, China) were added into each plate cell and ADCC lysis was stimulated. A Promega reagent (CytoTox 96, Promega, USA) was then added to test the LDH activity. EC_50_ was calculated from the correspondence between the absorbance value and the antibody concentration.

### *In Vivo* Pharmacology Assessments

#### Cell preparation

The Human leukemia cell line MC/CAR was purchased from ATCC (USA). Recommended media RPMI1640 supplemented with 10% fetal bovine serum was used as the culture medium. The cell culture volume was enlarged every 2 days and harvested for inoculation 8 days after resuscitation.

#### Subcutaneous tumor inoculation and treatment

SCID mice were purchased from Beijing Vital River Laboratory Animal Technology Co., Ltd. To generate a subcutaneous tumor model, 1e6 MC/CAR cells were injected into the flank of the mouse. When the average tumor volume reached 100 mm^3^, mice were stratified randomly according to tumor volume, with 10 mice in each group. Intraperitoneal injections of alemtuzumab, Mab-TH (0.1–30 mg/kg), and saline (untreated group) were then begun. Drug administration continued twice per week for 1 month. Tumor volume was recorded during the process.

#### Disseminated tumor inoculation and treatment

SCID mice were stratified randomly according to body weight, with 10 mice in each group. To generate a disseminated tumor model, they were injected intravenously with 1e6 MC/CAR cells. Alemtuzumab and Mab-TH (0.1–10 mg/kg, intravenous (i.v.)) were injected immediately and follow-up drug administration continued twice per week for 2 months. Intravenous injection of tumor cells resulted in the seeding of multiple organs, including the central nervous system, leading to hind limb paralysis and death. Kaplan–Meier survival analysis was performed using SPSS and the median survival time was calculated.

### *In Vivo* Toxicology Study

#### Animals and drug administration

The toxicology study in cynomolgus monkeys were performed at the National Shanghai Center for Drug Safety Evaluation and Research (subdivision of NMPA). A total of 48 male and female cynomolgus monkeys received from Guangxi Guidong primate Development Co., Ltd., were involved in repeated dose studies. The animals were aged 4–5 years and weighed 3–4 kg (female) or 4–5 kg (male). Since CD52 was expressed in erythrocytes in some cynomolgus monkeys and these animals would die from hemolysis (van der Windt et al., 2010) after Mab-TH administration, an altered Coombs test (de Giorgi et al., 1987) was conducted in all monkeys to ensure negative CD52 expression in erythrocytes.

The study was done in compliance with the International Council for Harmonization (ICH) guidance S6 “Preclinical Safety Evaluation of Biotechnology-Derived Pharmaceuticals” and good laboratory practice (GLP) regulations and the principles published by the NMPA. Animals were housed individually in stainless steel cages with an environment of 18–24°C, 40–70% humidity, a minimum of eight air changes per hour, and 12 hr dark–light cycle change.

For repeat dose study, 48 cynomolgus monkeys were randomly assigned into five groups to receive the vehicle, alemtuzumab (positive control, 3 mg/kg), or Mab-TH (1, 3, 10 mg/kg) triple per week for 4 weeks. The antibody was diluted in 200 mL saline and dripped intravenously for around 2 hr to avoid infusion reaction.

#### Regular clinical signs

The regular clinical signs, including mortality, abnormality, body weight, food consumption, and signs of unwellness were checked continually after drug administration. Detailed clinical assessments were conducted daily. Body weight was recorded weekly and food consumption was recorded daily for each animal. Except that, ophthalmic examinations were conducted prior to initiation of dosing and after 28 and 56 days of dosing.

#### Safety pharmacology evaluation

Safety pharmacological assays, including electrocardiographic examination, blood pressure, and respiratory frequency, were monitored twice before drug administration and three times after the first and final administration (15 min, 1 day, and 28 days after drug injection). These indicators would reflect whether the test substance would have adverse effects on the cardiovascular and respiratory system of the animals.

#### Hematology and biochemical evaluation

Blood samples for hematology and biochemical assays were drawn once in the adaptive phase, two times in the treatment period, and once in the withdrawal period. EDTA-anticoagulated whole blood was analyzed for complete blood counts using the ADVIA 2120 Hematology System (SIMENS, Germany), while uncoagulated serum was fluxed into a Hitachi-7060 Analytics System and Medical Easyplus ion analyzer (Hitachi, Japan) to measure the blood biochemical index. Indicators related to organ functions and immune functions (such as aspartate transaminase (AST), creatine kinase [CK], and immune cell counts) were particularly focused on.

#### Toxic kinetics assay

Serum drug concentration in toxicology study was measured using a sandwich enzyme-linked immunosorbent assay (ELISA) revamped from the method of Jilani et al. (2004). The target antibody was captured by goat antihuman IgG, and the target antibody bound was detected by horseradish peroxidase (HRP)-conjugated rabbit antihuman IgG detection antibody (Jackson Immuno Research Laboratories, USA). Results of serum analysis were used to calculate toxic kinetic parameters, such as peak concentration (*C*_max_) and area under curve (AUC_0__–__t_).

#### Anti-drug antibody and function neutralization

Antidrug antibody (ADA) formation was monitored every week after drug injection. A validated bridging-ELISA method (Thurmond et al., 1998) was used to determine the negative or positive status. Mab-TH antibody was coated to capture serum ADA, and another biotin-conjugated Mab-TH antibody was added as a second antibody. HRP-conjugated streptavidin was finally added as the chromogenic reagent. Samples from unrelated monkeys were measured to determine the baseline value, and tested samples whose values were over 1.099-fold were determined as positive.

Serum samples with positive ADA results were conducted to neutralization assay. The method was altered from the CDC method, in which whether serum samples could suppress the CDC effect of Mab-TH was investigated. The results were compared with blank serum, and tested samples that were remarkably lower than the blank serum (*p* < .05, *t*-test) were determined as positive.

#### Morphological and pathology evaluation

After 4 weeks of dosing, six animals in each group were euthanized for necropsy, and the rest of the animals were kept for another 4 weeks. Protocol-specified organs were examined and weighted. Tissues from the brain, various glands, digestive organs, heart, etc. were collected in modified Davidson's fixative and stored in 10% neutral-buffered formalin. These tissues were embedded in paraffin, sliced at 5 μM, stained with hematoxylin and eosin, and examined microscopically by several pathologists.

### Statistics

All analytical methods used in this study were validated prior to the measurement to ensure data accuracy and reproducibility. For the ELISA and *in vitro* activity assay, the reaction curve was fitted to a four-parameter logistic regression. All the samples were detected in duplicates, and the result was acceptable since the coefficients of variation (CV) were not more than 15%. Statistical analysis was performed using a two-sample equal variance (homoscedastic) *t*-test with Excel (Microsoft, Redmond, WA, USA). *p* < .05 was considered statistically significant.

## Results

### Cell Growth, Antibody Productivity, and Purity

TH6 cells (0.8e6 cells/ml) were inoculated into the reactor. The viable cell density reached its peak at the level of 35e6 cells/ml on Day 13. Then, the cell density decreased slightly during the perfusion period, with a maintained level of around 20e6 cells/ml. The maximal productivity reached over 0.3 g/l/day after Day 20, with a total batch yield of around 100 g from all the process. The productivity in this batch was lower than that in our previous study, but it was also higher than that of the fed batch (Zhuang et al., 2017).

Fewer aggregates were observed in Mab-TH than in alemtuzumab in our size exclusion chromatography-high performance liquid chromatography (SEC-HPLC) assay 0.9% versus 2.0%, indicating that a higher purity of antibody monomer was detected (99.1% vs. 98.0%). In cation exchange (CEX) analysis, the proportions of three components were identical (acid, main, and basic components, 20.8%, 73.0%, and 6.2% in Mab-TH vs. 21.6%, 74.4%, and 4.0% in alemtuzumab). These results showed that Mab-TH had high purity.

### *In Vitro* Pharmacology

To compare the binding affinity of Mab-TH to the antigen CD52, flow cytometry analysis was performed to determine the parameter. Table 1 shows the result that both mAbs generated a concentration–response curve with a half maximal effective concentration around 25 μg/ml. In the statistical analysis, none of the parameters differed significantly between the two mAbs (*p* > .05), thus validating their similar affinities.

**Table 1.** Comparison of *In Vitro* Pharmacology Properties of Mab-TH Versus Alemtuzumab

Binding assay	Product	Binding EC_50_ (μg/ml)	Relative activity (%)	*t*-test *p*-value
	Mab-TH	36.32, 24.89, 21.80	113.7, 92.2, 80.7	.85
	Alemtuzumab	31.94, 27.01, 27.01		
**CDC assay**	**Product**	**CDC EC_50_ (μg/ml)**	**Relative activity (%)**	***t*-test *p*-value**
	Mab-TH	7.40, 6.73, 5.61	115.6, 107.0, 100.2	.47
	Alemtuzumab	6.40, 6.29, 5.60		
**ADCC assay**	**Product**	**ADCC EC_50_ (μg/ml)**	**Relative activity (%)**	***t*-test *p*-value**
	Mab-TH	3.48, 4.49, 3.07	116.0, 106.9, 97.8	.70
	Alemtuzumab	3.00, 4.20, 3.14		

*Note*. EC_50_ = half maximal effective concentration; CDC =  complement-dependent cytotoxicity; ADCC = antibody-dependent cytotoxicity. Relative activity = Mab-TH EC_50_/alemtuzumab EC_50_.

CDC and ADCC will be induced after binding. In these experiments, the MC/CAR cell was used as the target cell by its steady expression of CD52. Both antibody products caused ADCC and CDC; the results in Table 1 show that there were no significant differences between the EC_50_ values in the three independent experiments (*p* > .05), indicating their similar *in vitro* pharmacological parameters.

### *In Vivo* Pharmacology

**Fig. 1.**
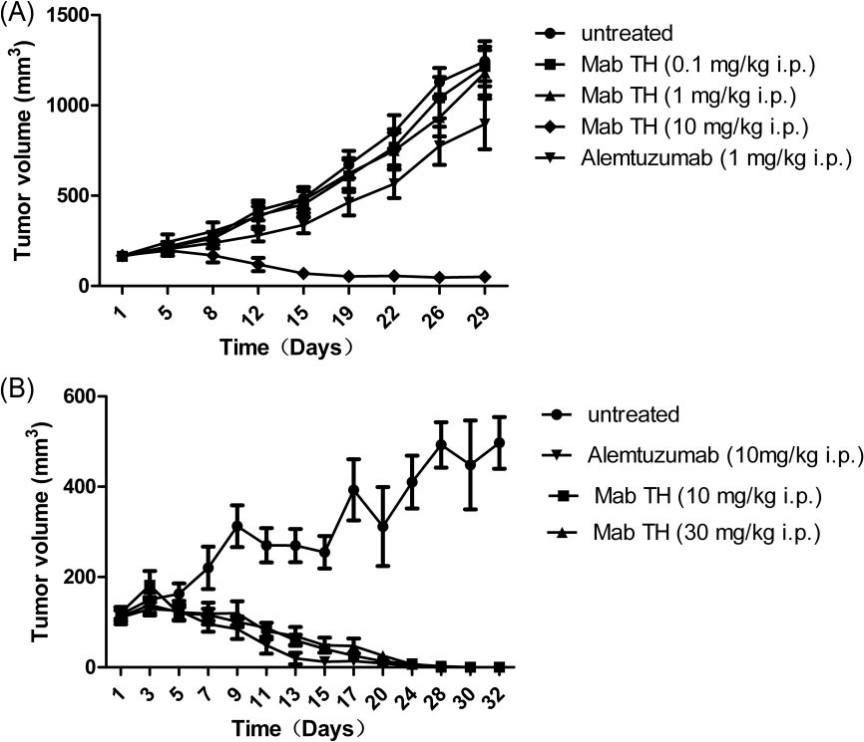
Activity of Mab-TH in subcutaneous tumor model. Mice were injected with MC/CAR cells and treated with low (A) or high (B) doses of Mab-TH and alemtuzmab. Tumor size was measured every 2–4 days. Mean tumor size ± MEM(Measurement Error Model) is shown for each time point.

The purpose of this study was to observe the effect of anti-CD52 mAb on transplanted tumor models of SCID mice. MC/CAR cells with high expression of CD52 were planted subcutaneous (s.c.) in SCID mice and treatment with Mab-TH up to 10 mg/kg intraperitoneal (i.p.) was first investigated. 0.1 and 1 mg/kg Mab-TH could not reverse the trend of tumor growth, while complete inhibition was observed when 10 mg Mab-TH was given (Fig. 1A). To confirm the results, another experiment with higher doses was implemented, in which 10 and 30 mg/kg Mab-TH i.p. eliminated the tumor effectively in 20 days (Fig. 1B). The result showed that the effective dose for both Mab-TH and alemtuzumab in tumor elimination was around 10 mg/kg.

**Fig. 2.**
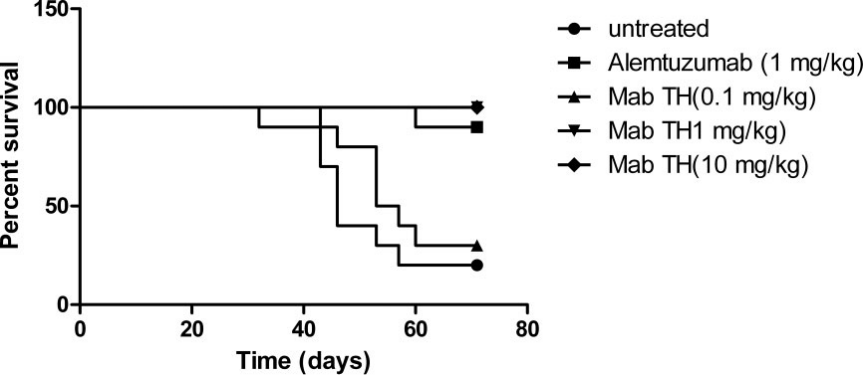
Survival plot of MC/CAR-disseminated severe combined immunodeficient mice. The groups included those receiving saline (untreated), alemtuzumab (1 mg/kg), and Mab-TH (0.1–10 mg/kg) twice per week for 2 months. Survival in all groups was maintained to 71 days. The alemtuzumab group and Mab groups over the dose of 1 mg/kg had significantly prolonged survival when compared with the untreated control group (*p* < .001).

For the disseminated tumor setting, MC/CAR cell lines were injected intravenously into the tail vein of SCID mice. Seeding of the tumor cells resulted in ataxia in the central nervous system, gave rise to hind limb paralysis, and finally caused death. Animals were observed daily and treatment with alemtuzumab or Mab-TH was initiated twice per week after tumor cell injection. The results showed that animals in the untreated group and the Mab-TH 0.1 mg/kg group died gradually after Day 30, reaching a final survival proportion of 20% and 30% (Fig. 2). Almost all the animals under the dose over 1 mg/kg survived to the end of the experiment (Fig. 2). Both alemtuzumab and Mab-TH provided a significant rise in survival rate in the disseminated tumor model.

### *In Vivo*, Repeat Dose Toxicology

CD52 antigen only expresses in the blood cells of primates, so it is not necessary to conduct toxicology experiments in rodents and dogs. Only the erythrocytes of humans, orangutans, and some cynomolgus monkeys do not express CD52, which can tolerate the effect of anti-CD52 mAb (van der Windt et al., 2010). Therefore, considering the feasibility, cynomolgus monkeys with negative CD52 expression in erythrocytes were selected into the groups.

In the clinical use of alemtuzumab, the maximum single dose of alemtuzumab was 30 mg/person, the frequency was once every other day, and the weekly cumulative dose was 90 mg/person. The main toxic reactions of alemtuzumab were hemocytopenia, lymphopenia, and infusion reaction. Our pre-experiment of mAb-TH also showed that the given dose of 3 mg/kg could lead to a decrease of peripheral erythroid cells, lymphocytes, T cells, and NK cells in cynomolgus monkeys. Based on the aforementioned information, the proposed doses in this study were 0, 1, 3, and 10 mg/kg, which are equivalent to 2, 6, and 20 times the clinical dose. Because this product is a biosimilar product, a single-dose toxicity test was not implemented.

#### Survival, clinical signs, body weight, and food consumption results

Of the 48 cynomolgus monkeys in the subgroups, none were found dead or *in articulo mortis*. No disorders in wellness signs, food consumption, and ophthalmic signs were observed. Body weight in female monkeys (10 mg/kg Mab-TH group) had a temporary reduction in the initial stage, but recovered since Day 14 (see Table 2). No body weight difference with the control group was found at the end of the experiment.

**Table 2. (:** A) Body Weight Statistics of Female Cynomolgus Monkeys in Toxicology Research; (B) Body Weight Statistics of Male Cynomolgus Monkeys in Toxicology Research

	Untreated	Mab-TH			Alemtuzumab
Time	0 mg/kg (*n* = 5)	1 mg/kg (*n* = 5)	3 mg/kg (*n* = 5)	10 mg/kg (*n* = 5)	3 mg/kg (*n* = 4)
(A)					
D1	3.5 ± 0.3	3.5 ± 0.3	3.4 ± 0.5	3.5 ± 0.3	3.3 ± 0.3
D7	3.4 ± 0.4	3.4 ± 0.4	3.3 ± 0.5	3.2 ± 0.3	2.7 ± 0.3^a^
D14	3.6 ± 0.3	3.5 ± 0.4	3.3 ± 0.5	3.4 ± 0.3	3.2 ± 0.2
D21	3.5 ± 0.3	3.5 ± 0.4	3.3 ± 0.4	3.5 ± 0.3	3.2 ± 0.2
D28	3.6 ± 0.3	3.5 ± 0.4	3.4 ± 0.5	3.5 ± 0.3	3.3 ± 0.2
	(*n* = 2)	(*n* = 2)	(*n* = 2)	(*n* = 2)	(*n* = 1)
R7	3.9 ± 0.1	3.8 ± 0.0	3.8 ± 0.1	3.8 ± 0.1	3.7
R14	3.8 ± 0.1	3.8 ± 0.1	3.7 ± 0.1	3.8 ± 0.2	3.5
R21	3.9 ± 0.1	3.8 ± 0.1	3.7 ± 0.0	3.8 ± 0.3	3.6
R28	4.0 ± 0.1	4.1 ± 0.4	4.0 ± 0.1	4.0 ± 0.2	3.9
(B)					
D1	4.6 ± 0.8	4.7 ± 0.9	4.4 ± 0.8	4.7 ± 0.5	4.3 ± 0.4
D7	4.6 ± 0.7	4.6 ± 0.9	4.2 ± 0.8	4.4 ± 0.5	3.9 ± 0.4
D14	4.6 ± 0.7	4.7 ± 0.9	4.3 ± 0.7	4.5 ± 0.4	4.1 ± 0.4
D21	4.6 ± 0.7	4.7 ± 0.9	4.5 ± 0.7	4.6 ± 0.3	4.3 ± 0.4
D28	4.7 ± 0.7	4.7 ± 0.9	4.5 ± 0.6	4.6 ± 0.4	4.3 ± 0.4
	(*n* = 2)	(*n* = 2)	(*n* = 2)	(*n* = 2)	(*n* = 1)
R7	5.1 ± 0.8	5.6 ± 0.6	4.9 ± 0.2	4.9 ± 0.0	4.8
R14	5.2 ± 1.0	5.6 ± 0.6	4.7 ± 0.2	4.7 ± 0.1	4.8
R21	5.1 ± 1.0	5.6 ± 0.7	4.8 ± 0.1	4.8 ± 0.1	4.8
R28	5.4 ± 1.1	5.8 ± 0.5	4.8 ± 0.1	4.9 ± 0.4	4.9

*Note*. “D” refers to days after first drug administration. ‘R’ refers to days after drug withdrawal.

^a^A significant difference from the control group (*t*-test, *p* < .05).

#### Hematology and biochemical analysis

**Fig. 3.**
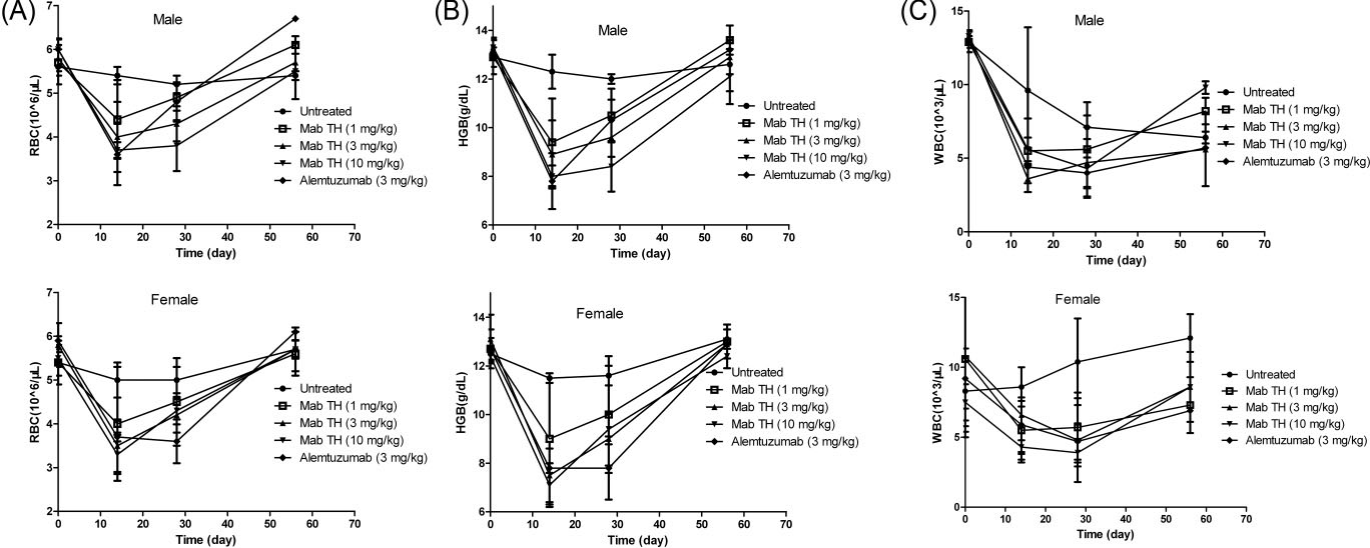
Changes of red blood cell counts (A), hemoglobin concentrations (B), and white blood cell counts (C) in male and female cynomolgus monkeys after the antibody dosing: filled circles, untreated group; open squares,1 mg/kg Mab-TH; upward triangles, 3 mg/kg Mab-TH; downward triangles, 10 mg/kg Mab-TH; and filled diamonds, 3 mg/kg alemtuzumab. Animal number was 5 for the untreated and Mab-TH groups and 4 for the alemtuzumab group before Day 28. Three animals from each group were sacrificed on Day 28 for pathology evaluation, and the remaining animals survived through Day 56.

As the antibody was mainly acted on hemocytes with CD52 expression, reduction in white blood cell (WBC) count was expected as a remarkable adverse reaction. The number of WBCs reduced to 40–60% of the baseline in 14–28 days in all dosing groups, but it reversed to the normal level after drug withdrawal (Fig. 3C). Another notable side effect was related to hypohemia. Red blood cell (RBC) count and RGB concentration declined on Day 14 in all treated groups, but recovered finally (Fig. 3A, B). Such a phenomenon was also previously reported (Keating et al., 2002), but the mechanism was not clarified.

**Fig. 4.**
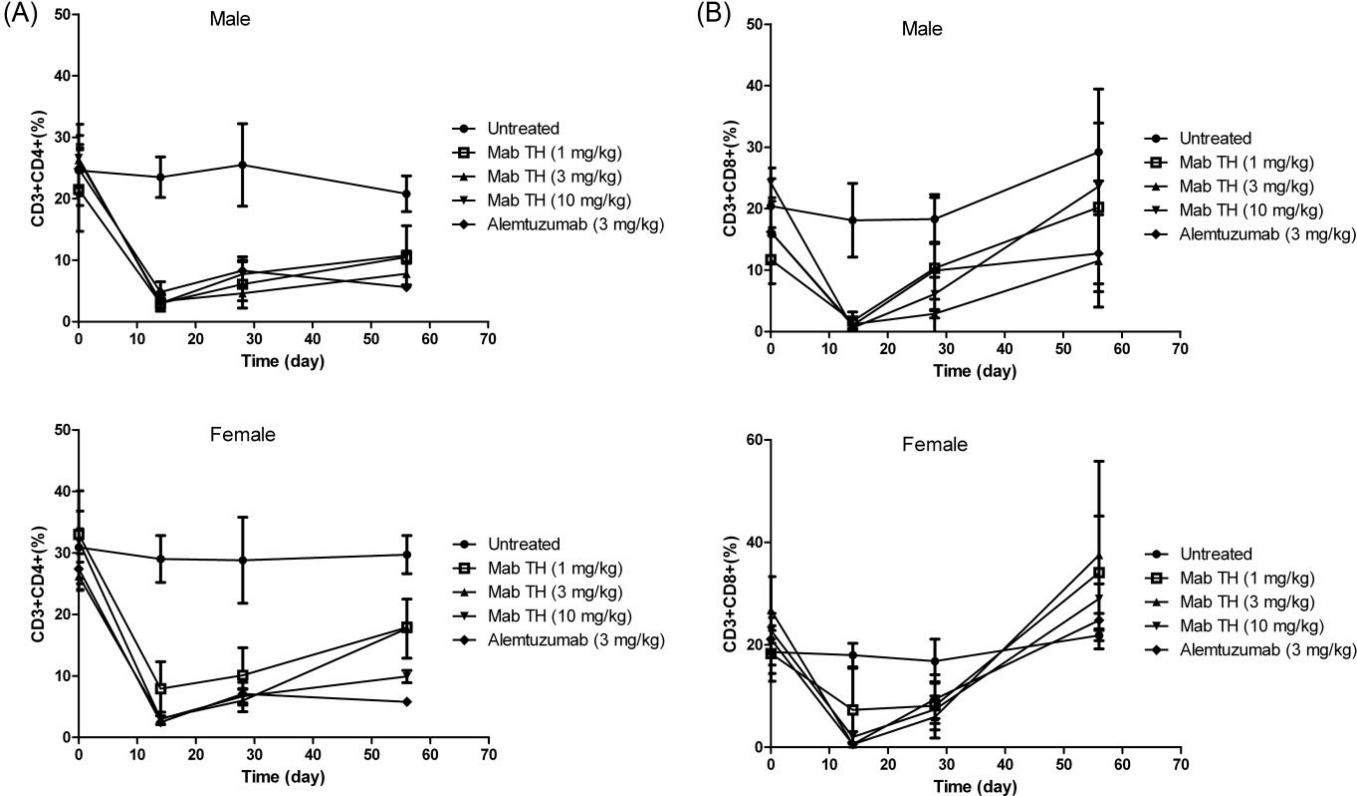
Changes of CD3 + CD4^+^ T cell proportions (A) and CD3 + CD8 + T cell proportions (B) in male and female cynomolgus monkeys after the antibody dosing: filled circles, untreated group; open squares, 1 mg/kg Mab-TH; upward triangles, 3 mg/kg Mab-TH; downward triangles, 10 mg/kg Mab-TH; and filled diamonds, 3 mg/kg alemtuzumab. Animal number was 5 for untreated and Mab-TH group and 4 for alemtuzumab group before day 28. Three animals from each group were sacrificed on day 28 for pathology evaluation, and the remaining animals survived through day 56.

To explore which subtype of immune cell would be infected by the anti-CD52 antibody, flow cytometry analysis was used to determine the change of proportion of some immune cells. The proportion of CD4+ and CD8+ T cells descended to a low level 2 weeks after drug administration. The proportion of CD8+ T cells recovered finally (Fig. 4B), while the CD4+ proportion could not return to its baseline (Fig. 4A). Such T cell reduction was entirely predicted as a pharmacodynamic result, and no difference was observed between Mab-TH and alemtuzumab.

No obvious biochemical changes occurred during the entire experiment duration, indicating the low toxicity of Mab-TH to unrelated viscera.

#### Toxic kinetic parameters and ADA formation

There were no sex-related differences in systemic exposure. Therefore, group mean toxicokinetic (TK) parameters are discussed using combined data, in which two indicators *C*_max_ and AUC were focused. *C*_max_ and AUC increased with the dose gradient in a proportional manner after first and last drug administration (Table 3). Mean exposure (AUC and *C*_max_) were higher on Day 27 relative to Day 1 with a ratio around 1.5–2.0, indicating some accumulation effect.

**Table 3.** Toxicokinetic Parameters of Mab-TH Following Weekly Intravenous Administration (3 Times per Week) of Mab-TH

Dose (mg/kg/dose)	Study day	*C*_max_ (µg/ml)	AUC_(0–t)_ (hr µg/ml)	AUC_(0–t)_/dose (hr kg/l)
1	1	12.82 ± 2.02	331.85 ± 163.06	414.81 ± 203.83
	27	19.68 ± 13.03	590.83 ± 549.81	738.54 ± 687.27
3	1	41.85 ± 6.03	1,386.17 ± 319.56	533.14 ± 122.91
	27	87.03 ± 18.33	2,523.41 ± 919.46	970.54 ± 353.64
10	1	164.99 ± 21.11	4,991.88 ± 1,052.87	499.19 ± 105.29
	27	257.68 ± 58.83	6,893.1 ± 3,310.79	689.31 ± 331.08
3 (alemtuzumab)	1	53.59 ± 8.24	1,746.99 ± 246.92	582.33 ± 82.31
	27	92.2 ± 37.46	2,725.52 ± 1684.67	908.51 ± 561.56

*Note*. All values are mean ± standard deviation. AUC_(0–t)_ is the area under the concentration–time curve from time 0 to the end of the experiment; *C*_max_ is the maximum drug concentration. Animals were dosed on Days 1–27, every two days. The last dose was on Day 27.

The results of ADA and neutralization activity formation are shown in Table 4. A general trend is that animals with high dose had an early formation of ADA (e.g., ADA appears in 10 mg/kg Mab-TH group as early as Day 7), and alemtuzumab produce ADA titer or neutralization activity earlier than Mab-TH in the same dose.

**Table 4.** Numbers and Subgroups of Animals With Positive Antidrug Antibody (ADA) or Neutralization Activity

Time (days)	ADA positive detected for the first time	Neutralization activity positive detected for the first time
7	1 animal in 10 mg/kg Mab-TH group	
28	2 animals in 3 mg/kg alemtuzumab group	2 animals in 3 mg/kg alemtuzumab group
42	3 animals in 3 mg/kg Mab-TH group	1 animal in 10 mg/kg Mab-TH group

*Note*. Animal numbers were 10 (5 male and 5 female) for the untreated and Mab-TH groups, and 8 (4 male and 4 female) for the alemtuzumab group before Day 28. Six animals (3 male and 3 female) from each group were sacrificed on Day 28 for pathology evaluation, and the remaining animals survived through Day 56.

#### Morphology and pathology results

To further evaluate the morphological and pathological impact of the drug, pathological evaluation was performed. In monkeys that survived until the study was terminated, noteworthy microscopic findings were all related to immune systems. Atrophy happened in tissues like the thymus gland, spleen, and mesentery nodes, which were rich in lymphocytes. In general, the incidence of these findings increased with increasing dose of Mab-TH or alemtuzumab, and the situation improved evidently during the drug withdrawal period (Table 5). Another remarkable pathology change was hematopoietic cell proliferation in marrow, which could be considered a compensation mechanism for the pharmacological effect.

**Table 5.** Main Pathology Findings at the End of the Treatment or the Whole Experiment

	Rates of pathological changes in each group			
Kind of pathological change	1 mg/kg Mab-TH	3 mg/kg Mab-TH	10 mg/kg Mab-TH	3 mg/kg alemtuzumab
Checkpoint: end of the treatment (D28)				
Atrophy of thymus gland	2/6	1/6	2/6	3/6
Lymphocytopenia in spleen	2/6	3/6	3/6	5/6
Lymphocytopenia in mesentery nodes	1/6	0/6	4/6	1/6
Hematopoietic cell proliferation in marrow	4/6	6/6	6/6	5/6
Checkpoint: end of the whole experiment (D56)				
Atrophy of thymus gland	0/4	1/4	3/4	0/2
Lymphocytopenia in spleen	0/4	0/4	0/4	0/2
Lymphocytopenia in mesentery nodes	0/4	0/4	0/4	0/2
Hematopoietic cell proliferation in marrow	0/4	1/4	2/4	0/2

Except for immune reaction, no other macroscopic findings were observed during the experiment, indicating that there were few irrelevant toxicities for Mab-TH.

## Discussion

Traditional anti-CD52 mAb production was performed through a fed-batch process. To increase the productivity and lower the cost, we developed a perfusion process, the product of which was coded as Mab-TH. In our previous study, perfusion and fed-batch processes were compared in the same culture conditions. The results showed that the batch productivity of the perfusion process was much higher than that of the fed batch, which supported perfusion being a more efficient method than the fed-batch process in the production of functional anti-CD52 mAb (Zhuang et al., 2017). Our previous research had also demonstrated that the perfusion process could lower the formation of aggregates, optimize the glycan feature, and elevate the CDC activity (Zhuang et al., 2017). This nonclinical research further investigated the quality of Mab-TH, showing that there were no biologically significant differences in pharmacological and safety effects.

Dose reaction curves from CD52 binding assay, CDC assay, and ADCC assay were tested for comparison of *in vitro* activity. The ratios of EC_50_ values between Mab-TH and alemtuzumab were calculated as relative activity. As for the criterion to biosimilar antibody, the relative activity should be controlled to 70–130%, in which results of Mab-TH compared with alemtuzumab from three independent determinations were satisfied.

Cure of transplanted tumor in SCID mice could offer an intuitive evaluation for antitumor antibodies. We repeated the experiment twice in the subcutaneous tumor model and confirmed that doses over 10 mg/kg i.p. were effective in tumor reduction. The results were similar to those of reported surveys (Zhang et al., 2003; Siders et al., 2010). Disseminated tumor transplant resulted in the seeding of tumor in multiple organs, including the central nervous system, leading to hind limb paralysis and finally death. Our survival curve results indicated a protective dose of 1 mg/kg for both Mab-TH and alemtuzumab, which could obviously extend the life span of transplanted SCID mice.

Although toxicology studies are not powered to access pharmacokinetic parameters, similar profiles were observed after 1 month of repeated dosing. Maximum plasma concentrations (*C*_max_) and AUC were basically similar for either product at identical dosages. Drug exposure increased with the dose gradient and the accumulation effect made *C*_max_ and AUC higher after the last dosing than their initial values. The pharmacokinetic behavior was also affected by the formation of ADA. Animals with positive ADA judgment would have a reduction in drug exposure, especially when the dosage exceeded 3 mg/kg (Table 6).

**Table 6.** Relationship Between the Reduction of AUC and the Formation of Antidrug Antibody (ADA)

	ADA positive after Mab-TH 3 mg/kg i.p.			ADA negative after Mab-TH 3 mg/kg i.p.						
Animal number	304	305	315	301	302	303	311	312	314	
AUC_(0–t)_ (hr µg/ml)	1,135.56	2,749.94	2,671.89	1,504.15	1,322.85	2,944.14	2,569.11	3,111.47	3,953.04	
	**ADA positive after Mab-TH 10 mg/kg i.p.**				**ADA negative after Mab-TH 10 mg/kg i.p.**					
Animal number	402	405	401	403	404	411	412	413	414	415
AUC_(0–t)_ (hr µg/ml)	1,295.65	2,385.11	4,329.49	8,814.47	7,212.14	10 573.50	11 121.34	8,696.77	8,173.20	6,329.34
	**ADA positive after alemtuzumab 3 mg/kg i.p.**				**ADA negative after alemtuzumab 3 mg/kg i.p.**					
Animal number	502	503	512	514	501	504	511	513		
AUC_(0–t)_ (hr µg/ml)	3,255.23	85.58	277.35	4,817.28	2,867.44	3,643.64	3,873.49	2,984.13		

In reports of clinical trials and applications of alemtuzumab, adverse events could be attributed to three parameters (Lin et al., 2005; Ranganathan et al., 2018; Thomas et al., 2016; Walsh et al., 2008), the infusion reaction (presumably caused by tumor cell lysis), the neutropenia and erythrocytopenia (mechanism was still unknown), and the bacteria or virus infection (consequence of immunosuppression). As the affinity of monkey CD52 to the antibody is much lower than that of human CD52, side effects in the preclinical research would be lighter than those in the clinical research. As expected, neither infection nor infusion reaction occurred in our preclinical study. The only related phenomenon was anemia and lymphocytopenia, in which the RBC counts and hemoglobin concentrations dropped to about 35% below the predosing baseline. Such adverse reaction was a transient response, and the status returned to normal in 1 month after drug withdrawal.

The anatomy and pathology report also offered no unexpected results. Only tissues with a high proportion of lymphocytes were atrophied as a result of lymphocyte depletion. The other finding was hyperplasia of bone marrow, which could be a kind of compensation response.

## Conclusion

Considering the results of our current and previous work together, we came to the conclusion that process optimization for the production of anti-CD52 mAb from fed batch to perfusion markedly improved the quality indicators, although no meaningful differences were observed in preclinical survey through *in vitro, in vivo*, and safety evaluations. Whether there will be some differences in clinical research awaits further investigation.
